# Correction: Automatized analysis of children’s exposure to child-directed speech in preschool settings: Validation and application

**DOI:** 10.1371/journal.pone.0248232

**Published:** 2021-03-08

**Authors:** The PLOS ONE Staff

In the title of this article, the word “reschool” should be “preschool.” The correct title is: Automatized analysis of children’s exposure to child-directed speech in preschool settings: Validation and application.

The third and fifth authors’ names are spelled incorrectly in the byline and in the author contributions. The correct names are: Leydi Johana Chaparro-Moreno and Kelly M. Purtell. The correct citation is: Gonzalez Villasanti H, Justice LM, Chaparro-Moreno LJ, Lin T-J, Purtell KM (2020) Automatized analysis of children’s exposure to child-directed speech in preschool settings: Validation and application. PLoS ONE 15(11): e0242511. https://doi.org/10.1371/journal.pone.0242511

The publisher apologizes for the error.
