# Supplementary material for: Automatized analysis of children’s exposure to child-directed speech in reschool settings: Validation and application
Source: PLoS One. 2020 Nov 25;15(11):e0242511. doi: 10.1371/journal.pone.0242511 (PMC7688182; doi:10.1371/journal.pone.0242511)
Supplement: Attachment — Submitted filename: PONE-D-20-15910_ReviewerComment-Rev3-Aug2020.pdf [file pone.0242511.s001.pdf]

REVIEWER COMMENTS

Submitted to: PLOS ONE

Title: "Automatized Analysis of Children's Exposure to Child-Directed Speech in Preschool Settings: Validation and Application"

Authors: Hugo Gonzalez Villasanti, Laura M. Justice, Leidy Johana Chaparro-Moreno, Tzu-Jung Lin, Kelly Purtell.

Clarity: Good

Correctness: Fair/Good

Implications: Good

Originality: Good

Recommendation: Revise/Update

General Comments:

This manuscript focuses on developing an automatic system to recognize child-adult and child-child interactions and CDS (child directed speech) in the preschool classroom environment. In this study, the primary subject (child) is referred to as the focal child and other children as referred to as peers. Research based on CDS has shown prior evidence that CDS might be an important factor to study the development of pre-school children's language. Although professional collection, recording, and evaluation of CDS is a time-intensive process, technological advances based on machine learning models might aid the transcription of the spoken language, and hereby reducing the time and effort in studying CDS. However, in this study, apart from using the spoken language content the authors have also leveraged visual feed from cameras mounted on the subjects' head.

So – there are two fundamental sides to the research study: (1) the ability to assess child-adult and child-child interactions and CDS (child directed speech) – which includes the types of speech/communication interaction concepts which are explored and if they reflect a realistic representation of child-directed speech; and (2) the speech recognition and audio plus video analysis technology used to estimate the needed raw data to see if these reflect the expected ground-truth observational data.

The subjects considered in this experiment were diverse in terms of race and socioeconomic status based on maternal education. The experimental recordings were collected twice in a day (morning, afternoon) and at two time-points in the academic year (fall, winter).

<u>COMMENT#1:</u> the amount of data recorded was very limited. In the original Hart and Risley study which was published as "Meaningful Differences" – they also had short 1hr recordings. To be effective here, much longer recordings which are in naturalistic context are needed. The proof-of-concept idea could be assessed with 1hr recordings, but from my experience with LENA analysis and speech recognition/analysis for diarization (who spoke what when and how), 3-8hr recordings would provide better overall measures that are more consistent long-term.

<u>COMMENT#2:</u> Line 73-25: yes, the LENA analysis software provides measures of Adult-word-count (AWC), child vocalizations (CV), and conversational turns (CT). The study ref's [10] was done by LENA foundation and they did benchmark on 1hr blocks but that was to assess the changes over the day. The original Hart & Risley study was more focused on the daily word count exposure for the child. Betty Hart's work emphasized the range of word exposure was important, and at least studies from LENA foundation folks showed (and in discussions with Todd Risley) was that the 1hr recorded analog cassette tapes in Hart's work over predicted what might take place in actual daily language rich environments. So – the question here is that these measures are all ESTIMATED from the audio stream. It would be helpful to bring this point out better here – since the text here suggests that the LENA software "extracts" this information vs. estimates it.

<u>COMMENT#3:</u> so, the publication [17] does point to some issues on accuracy of the LENA analysis software.

> acoustic environments and new developments in automatic speech recognition (16). Fourth, recent reviews raise concerns about the validity of the tool (17). As Cristia and colleagues demonstrate, there have been a number of validity studies examining performance of the LENA relative to hand-coding and transcription in early childhood settings, but many of these do not provide adequate data to compute agreement measures with reference values. While the

It is again important to understand how that system was developed and intended to be used. LENA was set up for 1 adult and 1 child alone in a room having conversation engagement. When speech is captured by LENA recorder in diverse spaces, the analysis software was not intended to be used for accurate analysis. There have been a number of studies on robustness of speech technology in naturalistic settings and how performance degrades in mismatch conditions (i.e., differences between training and testing conditions for audio streams when automatic acoustic/language models are trained). This study gives a number of examples where research laboratories thought they were characterizing a speech related concept, but instead the technology they used was not intended for use in that context:

[HTML] On the **issues** of **intra-speaker variability** and **realism** in speech, speaker, and language recognition tasks
JHL Hansen, H Bořil - Speech Communication, 2018 - Elsevier
Recent years have witnessed notable advancements in the areas of speech, speaker and language/dialect recognition. However, many of the emerging scientific principles appear to be drifting to the sidelines with the assumption that access to larger amounts of data is all that is required to address a growing range of issues relating to new scenarios. This study surveys several challenging domains in formulating effective solutions in realistic speech data, and in particular the notion of using naturalistic data to better reflect the potential …
[illegible]

Examples of studies that consider LENA in other languages:

[HTML] Correlation and agreement between Language ENvironment **Analysis** (**LENA**™) and manual transcription for Dutch natural language recordings
T Busch, A Sangen, F Vanpoucke… - Behavior research …, 2018 - Springer
… This allows **lena** to deal with the high variability of **speech** in early … We therefore chose an operationalization more similar to the concept of "utterances" as implemented in the popular Codes for the Human **Analysis** of Transcripts (chat)transcription system (MacWhinney, 2000 …
Cited by 21 Related articles All 6 versions

A concise protocol for the validation of Language ENvironment **Analysis** (**LENA**) conversational turn counts in Vietnamese
HV Ganek, A Eriks-Brophy - Communication Disorders …, 2018 - journals.sagepub.com
… Download article citation data for: A Concise Protocol for the Validation of Language ENvironment **Analysis** (**LENA**) Conversational Turn Counts in Vietnamese. Hillary V. Ganek, MA and Alice Eriks-Brophy, PhD. Communication Disorders Quarterly 2017 39:2, 371-380 …
Cited by 17 Related articles All 3 versions

Reliability studies (LENA foundation for the first entry here)

[PDF] Reliability of the **LENA** Language Environment **Analysis** System in young children's natural home environment
D Xu, U Yapanel, S Gray - Boulder, CO: **LENA** Foundation, 2009 - **lena**.org
… We have described the accuracy and reliability of **LENA** language environmental **analysis** software V3.1.0 in terms of segmentation, AWC estimates, and child vocalization … from the elimination of overlapping **speech**, and thus the **LENA** estimates were likely more …
Cited by 171 Related articles All 2 versions

[HTML] Reliability of the language environment **analysis** system (**LENA**™) in European French
M Canault, MT Le Normand, S Foudil… - Behavior research …, 2016 - Springer
… are absent in French. Although this should not play a decisive role in the processing performed by **LENA**, other acoustic **speech** cues may affect **LENA** automated procedures for vocal **analysis**. Differences in **speech** rate have …
Cited by 68 Related articles All 18 versions

Using the Language Environment **Analysis** (**LENA**) system in preschool classrooms with children with autism spectrum disorders
JR Dykstra, MG Sabatos-DeVito, DW Irvin, BA Boyd… - …, 2013 - journals.sagepub.com
… Download article citation data for: Using the Language Environment **Analysis** (**LENA**) system in preschool classrooms with children with autism spectrum disorders. Jessica R Dykstra, Maura G Sabatos-DeVito, Dwight W Irvin, Brian A Boyd, Kara A Hume, and Sam L Odom …
Cited by 81 Related articles All 10 versions

[HTML] Language ENvironment **analysis** (**LENA**) system investigation of day long recordings in children: A literature review
H Ganek, A Eriks-Brophy - Journal of Communication Disorders, 2018 - Elsevier
… Abstract. The Language ENvironment **Analysis** (**LENA**) System is a relatively new recording technology that can be used to … The purpose of this paper is to familiarize language acquisition researchers and **speech**-language pathologists with how the **LENA** System is …
☆ 99 Cited by 20 Related articles All 4 versions

I would agree that accuracy may not be consistent if the environmental context changes, but the text here should emphasize that the user therefore should use the technology only in the context it was intended, and if it's different – note that the user (not the software/LENA) is applying this tool/solution in a scenario which they should expect increased error. As a side note, LENA technology/software has been used in many countries outside of the USA, and applied to languages other than English, however all the software tools were developed using English data, and therefore it cannot be expected to work in that context.

The study considers three major experiments. First, facial features extracted using a computer vision service and speech activity extracted using an automatic speech recognition (ASR) service were used to predict instances of interactions of adults and peers in a preschool classroom with the focal children. Both the above services were offered in a pay-per-use format via Amazon Web Services. For this experiment, two bi-directional long-short term memory (BILSTM) classifiers were trained – one with high-level facial features and other with only face size, and both the models included speech activity features. Second, five word and level variables extracted using ASR were compared with the reference (ground truth). It should be note that the first two experiments were trained and validated only on Fall AM data. Third, the previously trained models using Fall AM data were deployed to predict the CDS variables in previously unseen data (Fall PM, Winter AM, Winter PM), thus examining children's interactions across a day and over time longitudinally.

<u>COMMENT#4:</u> so, it was surprising that the study used online ASR transcription support for the task here. The audio data is spontaneous and involves adult-child interaction, so it's not expected that ASR performance would be very good. These online services are intended to be used more for directed adult speech for maybe lectures, or telephony scenarios. The level of coarticulation seen in naturalistic data streams like this would not be a good choice. Also – it's not clear that the corresponding Lexicon and Language Model for the ASR engine (Amazon of course has extensive LM and Lexicon advancements), but the specifics here for adult-child interaction for me would be very different in terms of the needed performance. To benchmark the ASR tool – I might suggest having the adults actually read prepared text passages where groundtruth is known, and compare the ASR performance for this single speaker, clean/noise-free audio ASR output with what might have taken place in the 1hr recordings.

The first experiment indicated that the classifier trained using high-level facial features better predicted adult interactions while the reduced facial features predicted peer interactions

better. For the second experiment, the authors showed better performance for adult speakers compared to children, thereby denoting the lack of performance of transcribing child speech by current ASR services. They reported that the word error rate (WER) achieved by the ASR service is very high and hence not suitable. The analysis in the third experiment did not reveal significant differences on the five CDS variables. However, the authors depicted higher volume of adult speech during structured classroom task (Figure 2) and higher peer CDS and focal child speech during unstructured sessions (Figures 3 & 4).

COMMENT#5: A major limitation of this study, as reported by the authors (as well), is the automatic processing of the audio and video signals. Training the neural networks with features extracted from children or peers in this experiment, might improve the accuracy of child speech recognition models as compared to using the AWS neural networks. Being able to separate out the (i) performance of the tool, vs. (ii) the viability of the knowledge extracted in reflecting what is happening in groundtruth context is important. It is in this area where there needs to be more work to move this manuscript forward. I would suggest collecting some probe data with the same camera/audio capture setup, but in controlled setting with single adult speakers. Have them produce speech by (i) reading prepared text, and (ii) producing spontaneous speech. With video capture, the process could also simulate interaction by having a picture of a general child in a screen when the read or spontaneous speech should be directed. In this control context, it would allow the authors to benchmark the viability of the technology without the child in a controlled context. This would allow for a more effective comparison when used in natural scenario. I feel the existing analysis, while it could have been done on larger data streams, has value and I would not require they have to expand that effort.

Concluding Remarks: this is a interesting study which provides value in understanding child directed speech. The study in general is useful in contributing to the design of automatic Classroom Interaction Detection and Recognition (CIDR) systems. However, a lot of work is yet to be done to improve the neural network models or explore new features, and therefore achieve significant accuracy for using these CIDR systems as a reliable tool in CDS research. For the current effort, further benchmarking of the solution on controlled data is necessary to ensure consistency of the ASR and video technology with and without naturalistic context.

Some additional rewriting could help better place the impact of this study as it relates to other studies on language assessment via LENA software (as noted earlier).
